# Developing physical frailty specifications for investigation of frailty pathways in older people

**DOI:** 10.1007/s11357-016-9903-4

**Published:** 2016-04-08

**Authors:** Yew Y Ding

**Affiliations:** 1 Department of Geriatric Medicine & Institute of Geriatrics and Active Ageing, Tan Tock Seng Hospital, 11 Jalan Tan Tock Seng, Singapore, 308433 Republic of Singapore; 2 Department of Methodology, London School of Economics, WC2A 2AE, Columbia House, Houghton Street, London, UK

**Keywords:** Frailty, Specification, Aged, Frailty pathways, Validity

## Abstract

Different frailty definitions are suitable for different purposes. When investigating its key multidimensional predictors and effects, narrower definitions of frailty that exclude these elements may be more desirable. For this purpose, candidate physical frailty specifications are constructed and then evaluated on their construct and concurrent validity. For 4638 participants aged 65 to 89 years from wave 2 (2004) of the English Longitudinal Study of Ageing, confirmatory factor analysis is performed to create physical frailty specifications with four indicators (slowness, weakness, exhaustion, and weight loss) and with three indicators (slowness, weakness, and either exhaustion or weight loss). Using derived factor scores, their convergent, discriminant, and concurrent validity are compared. For specifications with four indicators and with three indicators including exhaustion, slowness contributes dominantly to the physical frailty factor. However, with three indicators including weight loss, weakness contributes most. Where represented, weight loss only contributes minimally. Higher factor scores are significantly associated with chronic diseases, functional impairment, and poor self-rated health, although less so for the third specification. Factor scores for the first two specifications have low correlation with psychological and social frailty while those for the third have negligible correlation. Factor scores increase with higher Frailty Index although again less so for the third specification. Minor differences are seen across gender. On account of their convergent, discriminatory, and concurrent validity, physical frailty specifications with four indicators and with three indicators including exhaustion hold promise for use in investigation of frailty pathways involving multidimensional predictors and effects.

**Electronic supplementary material:**

The online version of this article (doi:10.1007/s11357-016-9903-4) contains supplementary material, which is available to authorized users.

## Introduction

Frailty is widely regarded as the multidimensional loss of an individual’s reserves which results in vulnerability to developing adverse health-related outcomes (Espinoza 2005; Lally 2007; Pel-Littel 2009). It is conceptualized as the transitional state between robustness and functional decline (Lang 2009). The estimated prevalence of frailty is about 10 % among people aged 65 years living in the community (Collard 2012). Beyond mere numbers of affected people in any population, frailty is unfortunately associated with increased risk of death, disability, falls, hospitalization, and institutionalization (Daniels 2012; Ensrud 2009; Ensrud 2008; Jones 2005; Kiely 2009; Pilotto 2012; Woo 2012). Consequently, frailty plays a central role in determining the well-being of older people and has major public health importance (Woo 2006).

Over the past decade, the wide array of frailty instruments available (Pialoux 2012) attest to the absence of a universally accepted concept. Nevertheless, recent efforts to forge consensus among international experts have achieved some degree of agreement on suitable instruments for the recognition of frailty in older persons (Morley 2013). These instruments reflect different albeit overlapping concepts. Among them, two have gained greater prominence. The Cardiovascular Health Study (CHS) frailty phenotype is probably the most widely adopted. It conceptualizes frailty as a geriatric syndrome resulting from decline in multiple physiologic systems and is operationalized by requiring presence of at least three of its five components: shrinking (unintentional weight loss), weakness (low hand grip strength), poor endurance and energy (self-reported exhaustion), slowness (slow walking speed), and low physical activity level (based on self-report) (Fried 2001). The Frailty Index (FI) is possibly the second most widely applied instrument and is based on a deficit accumulation approach (Rockwood 2007b; Rockwood 2004). A count is taken of deficits which are a collection of symptoms, signs, diseases, disabilities, or test abnormalities. Selected deficits should be associated with poorer health status, should increase with age but not saturate too early, must as a group cover a range of systems, and must be the same for a group of people followed serially (Searle 2008). An increasing number of deficits raise the likelihood of being frail. It is expressed as the ratio of actual number of deficits to total possible number of deficits and is therefore a scalar measure ranging from 0 to 1. Besides these two instruments, the FRAIL tool was developed to identify older persons who are at risk for frailty. It consists of five self-reported items which are fatigue, resistance, ambulation, illnesses, and loss of weight. Presence of three or more items defines frailty (Morley 2013; van Kan 2008). In addition, the Tilburg Frailty Indicator (TFI) is based on an integral conceptual model of frailty which explicitly recognizes its multidimensional nature by defining losses in one of more of physical, psychological, and social functioning domains through its 15 items (Gobbens 2010a). It is scored from 0 to 15, with higher scores representing higher levels of frailty (Gobbens 2010b). While CHS frailty phenotype and FRAIL focus on the physical domain, FI and TFI attempt to measure frailty across more than a single domain.

Not surprisingly, all these four instruments predict future adverse outcomes in older people reasonably well (Fried 2001; Gobbens 2012; Rockwood 2007a; van Kan 2008). Moreover, where head-to-head comparisons are available, their predictive performance was shown to be approximately equivalent (Ravindrarajah 2013; Woo 2012). Past debate on which instrument is best among them appears to have run out its course over recent years and may now be less relevant in moving frailty research and public policy agendas forward. Rather, there is a growing sense that different instruments are best suited for different purposes (Cesari 2014; Martin 2008).

**Fig. 1:**
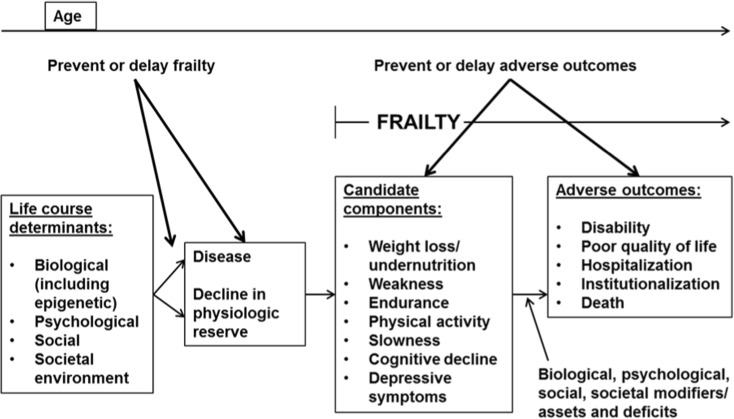
Working framework proposed by the Canadian Initiative on Frailty and Aging Frailty (adapted from Bergman 2004 with modifications)

Mapping of different frailty instruments to specific roles such as clinical screening, population studies, and biomedical research has been proposed (Bouillon 2013; Cesari 2014; Morley 2013). However, less work has been done in developing suitable specifications for investigating frailty pathways which represent relationships between frailty and its multidimensional predictors and effects. The working framework proposed by the Canadian Initiative on Frailty and Aging Frailty provides an excellent reference for this endeavor and is illustrated in Fig. 1. It conceptualizes frailty as having seven components including five items of the Frailty Phenotype and two additional items, namely, depression and impaired cognition (Bergman 2004). However, both depression and impaired cognition are psychological factors that could very well be represented as predictors and effects of frailty on its pathways. Having these as components of the frailty specification and at the same time as predictors or effects renders the task of teasing out their relationship with frailty very challenging. More recently, the integral concept of frailty proposed by Gobbens built on the Canadian framework. Here, frailty is explicitly specified as having separate physical, psychological, and social domains (Gobbens 2010a). Doing so allows physical frailty to be disaggregated from the other two frailty domains and in turn facilitates less constrained exploration of the relationship of frailty with multidimensional antecedents and effects. The adoption of this latter approach holds promise for developing frailty specifications that can be usefully applied when investigating relationships on these frailty pathways.

**Fig. 2:**
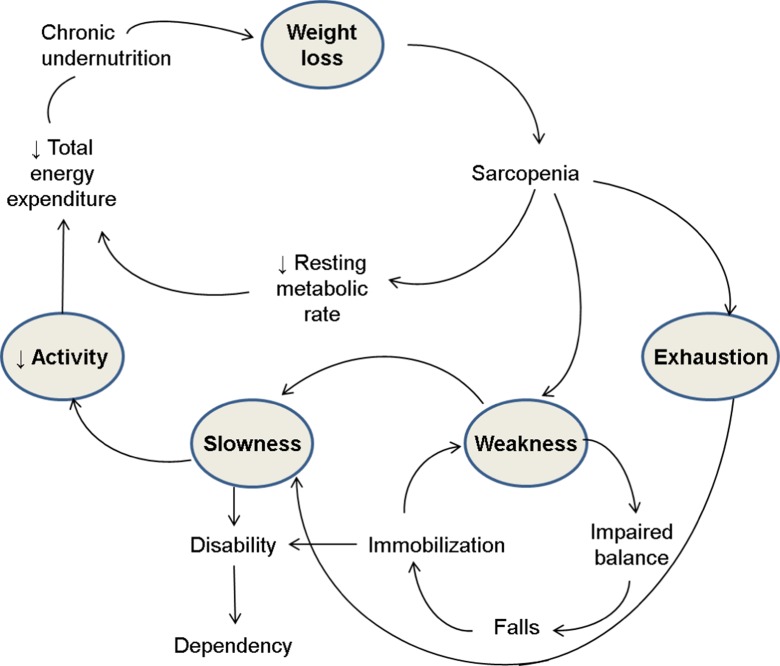
Modified representation of the cycle of frailty (adapted from Xue 2008)

In developing candidate physical frailty specifications for investigating frailty pathways, the CHS frailty phenotype provides a good starting point particularly as it is widely considered the prototype for physical frailty. Its conceptual framework is represented by the cycle of frailty in which its five components are positioned in a set of pathways (Xue 2008) as illustrated in Fig. 2. In this framework, sarcopenia holds a key position in the cycle of frailty in the sense that it is on the main pathway loop that includes weight loss, decreased total energy expenditure, and chronic undernutrition. Moreover, pathways emanating from it eventually lead to important health-related endpoints, namely, impaired balance, falls and injuries, immobilization, disability, and dependency. In these pathways to adverse outcomes, weakness and exhaustion are positioned immediately downstream to sarcopenia and slowness immediately follows weakness. Given this framework, it may be argued that among the five components of the CHS frailty phenotype, the cluster of exhaustion, weakness, and slowness appears central to the physical frailty concept and closest in proximity to its adverse outcomes. In addition, two of these five components pose interesting challenges. First, low physical activity is considered a predictor of frailty while its counter, exercise, is a modifier of frailty’s effect (Daniels 2008; Strawbridge 1998). On this account, it might be best excluded from the set of physical frailty indicators. Indeed, Buchman used the remaining four items to construct a composite measure of physical frailty albeit using body mass index instead of weight loss (Buchman 2009). Second, exhaustion may at times be a manifestation of depression in older people. In fact, two out of eight items of the Center for Epidemiologic Studies Depression (CES-D) scale were used to operationalize the component of exhaustion in the original study on the CHS frailty phenotype (Fried 2001). Moreover, depression resides within the psychological dimension and could itself be a potential target for interventions to reduce frailty and its effects. However, given that exhaustion is more typically related to physical conditions, it is unclear whether dropping it from the set of frailty indicators is necessary. With these points in mind, candidate physical frailty specifications based on the CHS frailty phenotype could omit physical inactivity and possibly exhaustion, thereby retaining three or four of the five original indicators.

Over this backdrop, the aims of this study are twofold. The first is to develop physical frailty specifications that are suitable for investigation of frailty pathways. These will be based on three or four components of the CHS frailty phenotype. The second is to evaluate and compare candidate specifications on their convergent, discriminant, and concurrent validity. The ultimate purpose is to obtain a frailty specification that can be used to quantify the relationships of frailty with its multidimensional predictors and effects. Ultimately, knowledge on these elements can inform broad strategies employing population-level interventions that seek to reduce frailty and its adverse effects in older people.

## Methods

### Data

Panel data from wave 2 (2004) of the English Longitudinal Study of Aging (ELSA) provides the requisite information. This is a longitudinal survey of a representative sample of the English population aged 50 years and older living in their homes at baseline (Steptoe 2013a). ELSA respondents aged 65 to 89 years at wave 2 are included. Those aged 90 years and older have their age merely coded as “90” and are thus excluded. All participants gave informed consent. Ethical approval for ELSA was granted by the Multicenter Research and Ethics Committee. Ethical oversight for this study is provided by procedures of the London School of Economics Ethics Policy.

### Measures

Indicators for physical frailty are based on four components of the CHS frailty phenotype (Fried 2001). Slowness is the average gait speed (m/s) of two attempts at walking a distance of 2.4 m multiplied by −1. Weakness is the dominant hand grip strength in kilograms multiplied by −1 for male and by −1.5 for female participants. The difference in expectation of grip strength mirrors population-independent cutoff values proposed for the CHS frailty phenotype criteria (Saum 2012). Weight loss is a binary variable for decrease in weight of more than 5 kg from wave 0 to 2. Weight at wave 0 is used as the reference because this was not measured at wave 1. Exhaustion is also a binary variable based on a positive reply to either or both of two items of the CES-D scale on whether the respondent “felt everything they did during the past week was an effort” and “could not get going much of the time in the past week” (Radloff 1977).

Using three permutations of these indicators, candidate physical frailty specifications are developed. The first specification has all four indicators, namely, slowness, weakness, exhaustion, and weight loss. Using latent class analysis of the CHS frailty phenotype, estimated probabilities of individual components for frail and non-frail states suggest that slowness and weakness discriminated best between them (Bandeen-Roche 2006). Thus, these two indicators are retained for remaining specifications. The second specification drops weight loss leaving the other three indicators. For the third, exhaustion instead of weight loss is dropped in view of the potential concerns already alluded to.

For psychological frailty, three indicators adapted from the TFI are constructed. First, impaired cognition is based on total cognitive index which combines test scores for memory and executive function which are recoded (0 to 49) so that higher scores indicate poorer function. Second, depressive symptoms are measured by a number of positive items in the CES-D scale. Given that the physical frailty indicator of exhaustion is based on two of its eight items, only the remaining six are used. Third, low resilience is measured in relation to three facets of adversity previously proposed (Demakakos 2008). Objective financial adversity is defined as being in the lowest quintile of total non-pension wealth. Self-perceived financial adversity is the report of sometimes or more often having too little money to spend on needs. Widowhood is the change of marital status from being married or single in wave 1 to being widowed in wave 2. The criterion for establishing resilience under these three facets of adversity is a CES-D score of three or less. Each facet is scored in an ordinal manner with “−1” if both adversity and resilience criteria are satisfied, “1” if only the criterion for adversity is satisfied, and “0” if only the criterion for resilience is satisfied or if neither criterion is satisfied. Summing up those for the three facets, a total score ranging from −3 to 3 is obtained where higher scores indicate lower resilience.

For social frailty, three indicators adapted from the TFI and based on previous work on social isolation (Steptoe 2013b) are constructed. First, loneliness is measured by the Revised UCLA Loneliness Score which comprises three items and scored from 3 to 9 (Hughes 2004). Second, poor social integration is a combination of five items (scored 0 to 15) on whether participants have no spouse and partner living with them, had little contact with children, had little contact with other family members, had little contact with friends, and were not a member of any organization, club, or society. Little contact was defined as less than monthly contact by meeting, phoning, or writing or e-mail. Third, poor social support is the combined score on three items (score 0 to 54) on whether there is lack of positive support, and occurrence of negative support. Lack of positive support is measured by positive answers to questions on whether children, other family members, and friends “criticizes the respondent,” “lets the respondent down,” and “gets on the nerves of respondent.” Negative support is measured by negative answers to questions on “understand the way you feel,” “can rely on if you had a serious problem,” and “can open up to them if you need to talk” with respect to children, other family members, and friends.

The FI (Rockwood 2007b; Rockwood 2004) is computed as the number of positive items out of 30 illnesses and functional impairments divided by 30 thereby deriving a scalar value of 0 to 1 (Searle 2008). In line with previously proposed cutoff values, people with FI of 0.08 or less are categorized as not frail, those with FI of 0.25 or more are frail, and the remaining are pre-frail (Song 2010).

### Statistical analyses

Confirmatory factor analysis (CFA) is performed in turn for the three candidate physical frailty specifications. Unique factor scores for each participant are derived for each specification. CFA is then repeated for subgroups defined by gender. Construct validity is assessed by considering convergent and discriminant validity. For convergent validity, functional impairment (number of basic activities of daily living (BADL) and instrumental activities of daily living (IADL) performed with difficulty), comorbidity (number of chronic illnesses), and poor self-rated health are regressed in turn on physical frailty factor scores (Rockwood 2005). Coefficients of the latter adjusting for age are obtained. For discriminant validity, Pearson’s coefficient is used to quantify the correlation of physical frailty factor scores with those of psychological and social frailty factors. Factor scores for the latter two are derived by CFA using their respective indicators. Pearson’s coefficient higher than 0.90 indicates very high correlation, 0.71 to 0.90 indicates high correlation, 0.51 to 0.70 indicates moderate correlation, 0.31 to 0.50 indicates low correlation, and 0.30 indicates negligible correlation (Hinkle 2003). To assess concurrent validity, FI is regressed on physical frailty factor scores and their coefficients adjusting for age are examined. For all validity checks, analyses are performed for the whole group and then in subgroups defined by gender.

CFA is performed with Mplus version 7.4 (Muthén & Muthén, 1998-2010) using maximum likelihood estimation with robust standard errors (MLR) which handles missing values by implementing full information maximum likelihood (FIML). MLR is selected over weighted least squares with mean and variance adjustment (WLSMV) because of better handling of missing values. For other regression analyses, missing values are handled by multiple imputation using chained equations to generate 20 sets. In using FIML and multiple imputation, the assumption of missing at random (MAR) is held. All other data analyses are performed with Stata version 13.1. Statistical significance is taken at *p* value of less than 0.05.

## Results

Data of 4638 people (2070 male and 2568 female) aged from 65 to 89 years are analyzed. Their characteristics are summarized in Table 1. They have on average two chronic illnesses. More than one quarter of them have some degree of functional impairment measured by basic activities of daily living. As expected, physical performance measured by walking speed and hand grip strength is worse among female participants. Frailty measured by both modified CHS frailty phenotype and Frailty Index is more common among them too. Thus, a significant proportion of participants have health issues and functional limitations. Among psychological measures, female participants display less resilience. Somewhat surprisingly, there are only minimal differences in social measures across gender.

**Table 1:** Characteristics of English Longitudinal Study of Aging (ELSA) wave 2 respondents aged 65 to 89 years included for analyses (*N* = 4638 for all, 2070 for male, and 2568 for female unless indicated otherwise)

Variables	All	By gender	
		Male	Female
General:			
Mean age, years (SD)	74.0 (6.3)	73.5 (6.2)	74.3 (6.4)
Female, *n*/*N* (%)	2568/4638 (55.4)	–	–
Mean chronic disease count (SD)	1.9 (1.4)^1^	1.8 (1.4)^2^	2.0 (1.4)^3^
Number of basic activities of daily living			
(BADL) items with difficulty, *n* (%)			
0	3389/4635 (73.1)	1560/2070 (75.4)	1829/2565 (71.3)
1 or 2	948/4635 (20.5)	389/2070 (18.8)	559/2565 (21.8)
3 or 4	220/4635 (4.8)	95/2070 (4.6)	125/2565 (4.9)
5 or 6	78/4635 (1.7)	26/2070 (1.3)	52/2565 (2.0)
Number of instrumental activities of daily living (IADL) items with difficulty, *n*/*N* (%)			
0	3308/4635 (71.4)	1593/2070 (77.0)	1715/2565 (66.9)
1 or 2	991/4635 (21.4)	358/2070 (17.3)	633/2565 (24.7)
3 or 4	236/4635 (5.1)	77/2070 (3.7)	159/2565 (6.2)
5 or 6 or 7	100/4635 (2.2)	42/2070 (2.0)	58/2565 (2.3)
Self-rated health, *n*/*N* (%)			
Excellent or very good	1554/4565 (34.0)	674/2029 (33.2)	880/2536 (34.7)
Good	1528/4565 (33.5)	686/2029 (33.8)	842/2536 (33.2)
Fair or poor	1483/4565 (32.5)	669/2029 (33.0)	814/2536 (32.1)
Physical:			
Mean average walking speed, m/s (SD)	0.82 (0.28)^4^	0.86 (0.27)^5^	0.78 (0.28)^6^
Hand grip strength (dominant hand), kg (SD)	25.9 (10.2)^7^	33.4 (8.9)^8^	19.6 (6.1)^9^
Exhaustion, *n*/*N* (%)	1490/4510 (33.0)	568/1997 (33.0)	992/25103(33.0)
Weight loss >5 kg from waves 0 to 2, *n*/*N* (%)	587/3590 (16.4)	255/1608 (15.9)	332/1982 (16.8)
Frailty status by modified CHS frailty phenotype, *n*/*N* (%)			
Not frail	866/3242 (26.7)	485/1462 (33.2)	381/1780 (21.4)
Pre-frail	1758/3242 (54.2)	775/1462 (53.0)	983/1780 (55.2)
Frail	618/3242 (19.1)	202/1462 (13.8)	416/1780 (33.4)
Frailty status by Frailty Index ^a^, *n*/*N* (%)			
Not frail	1444/3647 (39.6)	774/1639 (47.2)	670/2008 (33.4)
Pre-frail	1486/3647 (40.8)	629/1639 (38.4)	857/2008 (42.7)
Frail	717/3647 (20.7)	236/1639 (24.4)	481/2008 (33.9)
Psychological:			
Mean cognitive impairment score (SD)	18.9 (6.5)^10^	19.3 (6.4)^11^	18.5 (6.5)^12^
Mean CESD-8 ^c^ score (SD)	1.7 (2.0)^13^	1.3 (1.7)^14^	1.9 (2.1)^15^
Mean low resilience score ^d^ (SD)	0.20 (0.83)^16^	0.13 (0.80)^17^	0.26 (0.84)^18^
Social:			
Loneliness score ^e^ (SD)	4.2 (1.5)^19^	4.0 (1.4)^20^	4.3 (1.6)^21^
Mean poor social support score ^f^ (SD)	13.7 (7.0)^22^	14.7 (7.0)^23^	12.9 (6.8)^24^
Mean poor social integration score ^g^ (SD)	6.6 (2.5)^25^	6.7 (2.6)^26^	6.5 (2.5)^27^
Outcome:			
2-year mortality, *n*/*N* (%)	278/4638 (6.0)	147/2070 (7.1)	131/2568 (5.1)

*n* = ^1^4608, ^2^2052, ^3^2556, ^4^4092, ^5^1826, ^6^2266, ^7^3869, ^8^1760, ^9^2109, ^10^4348, ^11^1945, ^12^2403, ^13^4479, ^14^1987, ^15^2492, ^16^3854, ^17^1946, ^18^2460, ^19^3854, ^20^1746, ^21^2106, ^22^3339, ^23^1529, ^24^1810, ^25^3267, ^26^1506, and ^27^1761

^a^Frailty Index: 30 items (score 0 to 1)

^b^Cognitive impairment score: score 0 to 49

^c^CESD-8: Center for Epidemiologic Studies Depression Scale - 8 items (score 0 to 8)

^d^Low resilience score: 3 items (score 3 to 12)

^e^Loneliness score: Revised UCLA Loneliness Score - 3 items (score 3 to 9)

^f^Low social support score: 18 items (score 0 to 54)

^g^Poor social integration score: 6 items (score 0 to 15)

Table 2 summarizes the results of CFA for the three physical frailty specifications. For four indicators, slowness contributes most to the physical frailty factor whereas weakness and exhaustion do so to a lesser extent. Weight loss contributes much less. Similarly for three indicators including exhaustion, slowness contributes more than weakness and exhaustion do. On the other hand, for three indicators including weight loss, weakness contributes most with slowness doing so less. Here again, weight loss contributes minimally. These patterns are generally consistent across gender with only minor differences seen. These factor loading patterns reflect the stronger correlation among slowness, weakness, and exhaustion, compared with their correlation with weight loss. These differences in correlation are supported by tetrachoric correlation coefficients of weight loss with the other three indicators (0.16 to 0.19) being much lower than those between the other three (0.29 to 0.43) for the whole group as shown in Table S6 of the Supplementary Materials. Histograms showing approximately normal distribution of derived factor scores for the three physical frailty specifications are provided in Fig. S3 of the Supplementary Materials.

**Table 2:** Measurement model for three specifications of physical frailty and using confirmatory factor analysis (CFA) using full information maximum likelihood (FIML) [*N* = 4547 (all), 2019 (male), and 2528 (female) for the first two specifications; *N* = 4440 (all), 1985 (male), and 2455 (female) for the third specification]

Physical frailty specification	Standardized coefficient (standard error)		
	All	By gender	
		Male	Female
Four indicators:			
Slowness ^a^	0.76 (0.03)	0.73 (0.05)	0.77 (0.03)
Weakness ^b^	0.54 (0.02)	0.47 (0.04)	0.56 (0.03)
Exhaustion ^c^	0.55 (0.02)	0.56 (0.04)	0.54 (0.03)
Weight loss ^d^	0.28 (0.03)	0.24 (0.06)	0.31 (0.04)
Three indicators including exhaustion:			
Slowness ^a^	0.78 (0.03)	0.79 (0.05)	0.78 (0.04)
Weakness ^b^	0.52 (0.02)	0.79 (0.05)	0.55 (0.03)
Exhaustion ^c^	0.54 (0.03)	0.53 (0.04)	0.54 (0.03)
Three indicators including weight loss:			
Slowness ^a^	0.60 (0.05)	0.45 (0.08)	0.68 (0.06)
Weakness ^b^	0.68 (0.05)	0.76 (0.13)	0.63 (0.06)
Weight loss ^d^	0.30 (0.03)	0.26 (0.06)	0.33 (0.04)

*p* values are <0.05 for all coefficients

^a^Slowness: mean gait speed multiplied by a factor of −1

^b^Weakness: dominant hand grip strength multiplied by a factor of −1 (males) or −1.5 (females)

^c^Exhaustion: positive response to either or both of two items of CES-D scale on “could not get going much of the time in the past week” and “felt everything they did during the past week was an effort”

^d^Weight loss: decrease in weight of more than 5 kg from wave 0 to wave 2

In terms of convergent validity, Table 3 shows that for 1 standard deviation (SD) increase in the physical frailty factor score, the number of chronic diseases increases by 0.28 to 0.35 across specifications and gender after adjusting for age. Similarly, for 1 SD increase in physical frailty factor score, the combined number of items of BADL and IADL performed with difficulty increases by 0.36 to 0.51. Finally, for 1 SD increase in physical frailty factor score, the number of categories of poor self-rated health increases by 0.38 to 0.54. Regression coefficients are of similar magnitude across gender with the exception of the specification with three indicators including weight loss where they are clearly higher for female participants. Overall, regression coefficients for physical frailty factor scores with four indicators and with three indicators including exhaustion are very similar. However, their coefficients are higher than those with three indicators including weight loss, particularly for prediction of BADL and ADL difficulties, as well as poor self-rated health where there is minimal or no overlap of confidence intervals. The likely explanation is that as individual indicators, slowness, weakness, and exhaustion predict these outcomes better than weight loss does. This is supported by the results of linear regression analyses shown in Table S7 of the Supplementary Materials which show that slowness and exhaustion predict these three outcomes best, followed distantly by weakness, and finally weight loss. Notably, Akaike information criterion (AIC) and Bayesian information criterion (BIC) values for models with the first two specifications are similar while those with the third specification are higher (see footnote of Table 3). This means that the goodness of fit of models with the first two specifications are similar but better than those with the third specification. In addition, *r*-square values and, therefore, the variance explained for models with the first two specifications are also very similar but higher than those with the third specification (see footnote of Table 3). Overall, these findings indicate that the first two physical frailty specifications have higher convergent validity than the third.

**Table 3:** Linear regression of chronic disease, functional status, and self-rated health on factor scores for three physical frailty specifications adjusted for age: standardized coefficients (95 % confidence interval) (*N* = 4638 for all, 2070 for male, and 2568 for female)

	All	By gender	
		Male	Female
Number of chronic diseases:			
4 indicators	0.35 (0.32 to 0.38)^a^	0.34 (0.29 to 0.38)	0.34 (0.30 to 0.39)
3 indicators (including exhaustion)	0.34 (0.31 to 0.37)^b^	0.33 (0.29 to 0.38)	0.34 (0.30 to 0.38)
3 indicators (including weight loss)	0.30 (0.27 to 0.33)^c^	0.28 (0.23 to 0.33)	0.29 (0.25 to 0.34)
Number of basic and instrumental activities of daily living items performed with difficulty:			
4 indicators	0.49 (0.46 to 0.52)^d^	0.46 (0.42 to 0.50)	0.51 (0.47 to 0.55)
3 indicators (including exhaustion)	0.48 (0.46 to 0.51)^e^	0.45 (0.41 to 0.50)	0.50 (0.46 to 0.54)
3 indicators (including weight loss)	0.39 (0.36 to 0.43)^f^	0.36 (0.31 to 0.41)	0.41 (0.37 to 0.45)
Categories of poor self-rated health:			
4 indicators	0.51 (0.48 to 0.53)^g^	0.48 (0.43 to 0.52)	0.54 (0.51 to 0.58)
3 indicators (including exhaustion)	0.50 (0.47 to 0.53)^h^	0.47 (0.43 to 0.51)	0.53 (0.50 to 0.57)
3 indicators (including weight loss)	0.40 (0.37 to 0.43)^i^	0.38 (0.33 to 0.43)	0.44 (0.40 to 0.48)

*p* values are <0.05 for all coefficients

^a^AIC/BIC = 15,902/15,922, *r* ^2^ = 0.12

^b^AIC/BIC = 15,917/15,935, *r* ^2^ = 0.12

^c^AIC/BIC = 16,071/16,090, *r* ^2^ = 0.09

^d^AIC/BIC = 18,572/18,591, *r* ^2^ = 0.25

^e^AIC/BIC = 18,600/18,620, *r* ^2^ = 0.25

^f^AIC/BIC = 19,048/19,067, *r* ^2^ = 0.17

^g^AIC/BIC = 13,018/13,038, *r* ^2^ = 0.23

^h^AIC/BIC = 13,047/13,066, *r* ^2^ = 0.22

^i^AIC/BIC = 13,483/13,503, *r* ^2^ = 0.14

For discriminant validity, Table 4 shows that physical frailty factor scores for the first two specifications have low correlation with psychological frailty and negligible correlation with social frailty. In contrast, physical frailty factor score for the third specification has negligible correlation with the other frailty domains, suggesting higher discriminant validity. Nevertheless, all Pearson’s coefficients are well below the arbitrary 0.85 cutoff level where greater values are regarded as indicating low discriminant validity. Equally important, the correlation coefficients between the three different physical frailty specifications (multimethod) are in the region of 0.88 to 1.00 and therefore much larger than those between physical frailty and psychological frailty or social frailty (multitrait) which are from 0.12 to 0.41. This further supports discriminant validity. The corresponding Modified Multitrait Multimethod (MTMM) Matrix is provided in Table S8 of the Supplementary Materials.

**Table 4:** Correlation of factor scores for three physical frailty specifications with those for psychological and social frailty, and between factor scores for three physical frailty specifications (modified multitrait multimethod analysis): Pearson’s coefficient (95 % confidence interval) (*N* = 4638 for all, 2070 for male, and 2568 for female)

	Pearson’s correlation coefficient (Standard error)		
	All	By gender	
		Male	Female
Between physical and psychological frailty:			
4 indicators	0.41	0.38	0.40
	(0.38 to 0.43)	(0.34 to 0.42)	(0.36 to 0.43)
3 indicators (including exhaustion)	0.40	0.38	0.39
	(0.37 to 0.43)	(0.34 to 0.42)	(0.35 to 0.43)
3 indicators (including weight loss)	0.29	0.26	0.28
	(0.26 to 0.32)	(0.22 to 0.30)	(0.24 to 0.32)
Between physical and social frailty:			
4 indicators	0.16	0.18	0.17
	(0.13 to 0.20)	(0.14 to 0.23)	(0.12 to 0.22)
3 indicators (including exhaustion)	0.16	0.18	0.17
	(0.13 to 0.19)	(0.13 to 0.23)	(0.12 to 0.21)
3 indicators (including weight loss)	0.12	0.13	0.14
	(0.08 to 0.15)	(0.08 to 0.18)	(0.09 to 0.18)
Between specifications of physical frailty:			
3 indicators (including exhaustion) and 3 indicators (including weight loss)	0.89	0.88	0.89
	(0.87 to 0.90)	(0.86 to 0.90)	(0.87 to 0.90)
4 indicators and 3 indicators (including exhaustion)	1.00	1.00	1.00
	(0.99 to 1.00)	(0.99 to 1.00)	(0.99 to 1.00)
3 indicators (including weight loss) and 4 indicators	0.90	0.90	0.90
	(0.89 to 0.91)	(0.88 to 0.91)	(0.89 to 0.92)

*p* values are <0.05 for all correlation coefficients

Where concurrent validity is concerned, multiple linear regression analyses obtained statistically significant coefficients for all three physical frailty specifications as shown in Table 5. For 1 SD increase in physical frailty factor scores, the FI increases by 0.61 to 0.76 SD. There are minor variations of regression coefficients across gender. Overall, regression coefficients are higher for specifications with four indicators and with three indicators including exhaustion than those for the third specification. Yet again, AIC and BIC values for models with the first two specifications are similar while those with the third specification are higher. *R*-square values are almost equivalent for the first two specifications and higher than those of the third (see footnote of Table 5). This means that the goodness of fit of models and variance explained by models with the first two specifications are similar but better or higher than those with the third specification. Together, these findings indicate that concurrent validity for the first two specifications is higher than that for the third.

**Table 5:** Linear regression of Frailty Index on factor scores for three physical frailty specifications adjusted for age with multiple imputation: standardized coefficients (*N* = 4638 for all, 2070 for male, and 2568 for female)

	All	By gender	
		Male	Female
4 indicators	0.76 (0.73 to 0.79) ^a^	0.76 (0.71 to 0.80)	0.76 (0.72 to 0.79)
3 indicators (including exhaustion)	0.75 (0.72 to 0.78) ^b^	0.74 (0.70 to 0.79)	0.74 (0.71 to 0.78)
3 indicators (including weight loss)	0.62 (0.59 to 0.65) ^c^	0.61 (0.55 to 0.66)	0.61 (0.57 to 0.65)

*p* values are <0.05 for all physical frailty factor scores coefficients

^a^AIC/BIC = −6998/−6978, *r* ^2^ = 0.44

^b^AIC/BIC = −6918/−6899, *r* ^2^ = 0.43

^c^AIC/BIC = −5977/−5957, *r* ^2^ = 0.30

As sensitivity analysis, the CFA for physical frailty and regressions for evaluating convergent and concurrent validity are repeated using the WLSMV estimator. Comparison of coefficients obtained using MLR and WLSMV estimators is provided in Tables S9, S10, and S11 in the Supplementary Materials. Overall, only trivial differences are observed in the coefficients which do not change the interpretation of the results.

## Discussion

This study reports the development of frailty specifications for the purpose of investigating multidimensional predictors and effects of frailty such as those proposed by the working framework of the Canadian Initiative on Frailty and Aging. Rather than adopting a broad definition, narrower focus on physical frailty is employed to enhance prospective application when investigating its relationship with multidimensional elements on frailty pathways. In addition, physical frailty is viewed as a construct and is thus developed as a factor. Unlike the case with established frailty instruments including the CHS frailty phenotype where contribution of separate components is arbitrarily assumed and then fixed, latent variable analysis through CFA is performed to empirically derive the relationship of each indicator with the physical frailty factor. Furthermore, CFA allows measurement error to be accounted for, which is particularly relevant as performance measures are used as indicators. This combined approach represents an advance on the frailty specification proposed in the working framework of the Canadian Initiative on Frailty and Aging and further builds on that put forth in the integral concept of frailty (Bergman 2004; Gobbens 2010a).

To begin with, content validity is retained to a large extent given that the selected indicators used are drawn from the original components of the CHS frailty phenotype which is still widely regarded as the prototype of physical frailty. Higher weightage is accorded to slowness, weakness, and exhaustion given the relative importance of their positions in the cycle of frailty. Thus, the physical frailty specification with three indicators including exhaustion could be considered as having the essential set of indicators. Furthermore, for candidate specifications examined, slowness is central to the physical frailty factor except for the case with three indicators including weight loss. Weight loss clearly contributes little and an argument may be made for its exclusion as an indicator at least on the basis of the findings from this study. It is notable that our results are generally consistent across gender. More crucially, higher convergent and concurrent validity with four indicators and with three indicators including exhaustion are demonstrated over the third specification. Although the latter performs better on discriminant validity, the first two specifications have sufficiently low correlation with psychological and social frailty to suggest that overlap of their constructs is probably not large enough to be of practical concern. This is important when examining the relationship of physical frailty with multidimensional elements including those which are closely related to or are themselves deployed as indicators of psychological and social frailty.

Given these findings, physical frailty specifications with four indicators and that with three indicators including exhaustion appear to be suitable candidates for use in investigation of frailty pathways. Minimal contribution of weight loss as the fourth indicator suggests that three indicators, namely, slowness, weakness, and fatigue may be sufficient to represent physical frailty. However, the performance of these physical frailty specifications in predicting adverse health-related outcomes needs to be separately evaluated. This is an issue that is addressed in further research.

Although encouraging, these findings should to be viewed in the context of the study limitations. First, data from only one population is used and, therefore, there is uncertainty on the extent to which these findings may be generalized to other populations. To address this issue, the measurement model from this study needs to be applied to data from other populations in future work. Next, secondary data is used. The consequence is that choice of variables representing physical frailty is inevitably restricted. Nevertheless, selected variables arguably have face validity. The major challenge is the quantification of weight loss which is by necessity across the span of 4 years due to weight measurements not being available in wave 1. It remains to be seen whether weight loss may perform better as an indicator when change is measured across a shorter period of time such as 2 years. Third, missing data may introduce bias in our analyses. Multiple imputation is used to handle this issue here and requires the missing at random (MAR) assumption. Notwithstanding the inevitable uncertainty on the extent of bias introduced, this is not likely to be large enough to change the conclusions on the validity of physical frailty specifications evaluated here.

On the other hand, the strengths of this study include the use of ELSA which offers representative, reliable, and high-quality data that has produced a wealth of information on how older people age in England. Moreover, our relatively large sample size allows greater precision in estimation. Lastly, availability of physical performance measures for two physical frailty indicators provides more detailed information than questionnaire data alone would.

In conclusion, narrowing of the frailty specification to that of physical frailty is argued on the grounds that multidimensional elements on frailty pathways are best excluded from the set of its indicators. Suitable indicators are drawn from components of the CHS frailty phenotype and include slowness, weakness, and exhaustion with or without weight loss. In addition to retaining face and content validity, these two physical frailty specifications have demonstrated reasonable convergent, discriminatory, and concurrent validity using the data of older people living in England. Together, they hold promise as physical frailty specifications to be applied in the investigation of frailty pathways.

## Electronic supplementary material

ESM 1 (DOCX 40 kb)
