# Supplementary material for: Developing physical frailty specifications for investigation of frailty pathways in older people
Source: Age (Dordr). 2016 Apr 8;38(2):47. doi: 10.1007/s11357-016-9903-4 (PMC5005901; doi:10.1007/s11357-016-9903-4)
Supplement: Supplementary file 1 — (DOCX 40 kb) [file 11357_2016_9903_MOESM1_ESM.docx]

**Supplementary Materials**

Table 6: Tetrachoric correlations between indicators for physical frailty specified as binary variables: coefficients (N)

|  | Slowness | Weakness | Exhaustion | Weight loss |
| --- | --- | --- | --- | --- |
| **All** | | | | |
| Slowness | 1.00 (4,092) |  |  |  |
| Weakness | 0.43 (3,530) | 1.00 (3,869) |  |  |
| Exhaustion | 0.43 (4,067) | 0.29 (3,844) | 1.00 (4,510) |  |
| Weight loss | 0.18 (3,325) | 0.19 (3,509) | 0.16 (3,572) | 1.00 (3,590) |
| **Male** | | | | |
| Slowness | 1.00 (1,826) |  |  |  |
| Weakness | 0.43 (1,604) | 1.00 (1,760) |  |  |
| Exhaustion | 0.41 (1,812) | 0.24 (1,742) | 1.00 (1,997) |  |
| Weight loss | 0.13 (1,484) | 0.22 (1,591) | 0.17 (1,595) | 1.00 (1,608) |
| **Female** | | | | |
| Slowness | 1.00 (2,266) |  |  |  |
| Weakness | 0.41 (1,926) | 1.00 (2,109) |  |  |
| Exhaustion | 0.43 (2,255) | 0.31 (2,102) | 1.00 (2,513) |  |
| Weight loss | 0.22 (1,841) | 0.17 (1,918) | 0.15 (1,977) | 1.00 (1,982) |

P-values are <0.05 for all coefficients

Binary variables for slowness and weakness are created by adopting a proposed modification of the Fried frailty criteria that uses population-independent cutpoints (Saum 2012).

Table 7: Linear regression of chronic disease, functional status, and self-rated health on four binary components of the CHS frailty phenotype adjusted for age with multiple imputation: standardized coefficients (95% confidence interval) [N=4,638 for all, 2,070 for male, and 2,568 for female]

|  | **All** | **By gender** | |
| --- | --- | --- | --- |
|  |  | **Male** | **Female** |
| **Number of chronic diseases:** | | | |
| Slowness | 0.71 (0.62 to 0.79) | 0.68 (0.55 to 0.82) | 0.70 (0.58 to 0.82) |
| Weakness | 0.37 (0.28 to 0.46) | 0.31 (0.16 to 0.46) | 0.38 (0.25 to 0.50) |
| Exhaustion | 0.74 (0.66 to 0.83) | 0.83 (0.69 to 0.96) | 0.66 (0.55 to 0.77) |
| Weight loss | 0.29 (0.16 to 0.42) | 0.32 (0.12 to 0.51) | 0.26 (0.55 to 0.77) |
| **Number of basic and instrumental activities of daily living items performed with difficulty:** | | | |
| Slowness | 1.40 (1.27 to 1.52) | 1.34 (1.16 to 1.52) | 1.42 (1.24 to 1.59) |
| Weakness | 0.78 (0.63 to 0.93) | 0.72 (0.52 to 0.93) | 0.79 (0.59 to 0.98) |
| Exhaustion | 1.65 (1.53 to 1.77) | 1.60 (1.43 to 1.78) | 1.66 (1.51 to 1.82) |
| Weight loss | 0.54 (0.29 to 0.79) | 0.48 (0.08 to 0.88) | 0.57 (0.30 to 0.84) |
| **Categories of poor self-rated health:** | | | |
| Slowness | 0.79 (0.72 to 0.86) | 0.81 (0.71 to 0.91) | 0.79 (0.70 to 0.88) |
| Weakness | 0.44 (0.37 to 0.52) | 0.42 (0.30 to 0.53) | 0.50 (0.40 to 0.60) |
| Exhaustion | 0.92 (0.86 to 0.99) | 0.94 (0.85 to 1.05) | 0.92 (0.84 to 1.00) |
| Weight loss | 0.34 (0.24 to 0.44) | 0.35 (0.19 to 0.50) | 0.33 (0.20 to 0.47) |

P-values are <0.05 for all coefficients.

Table 8: Modified Multitrait Multimethod Matrix for physical, psychological, and social frailty: Pearson’s coefficients for the whole group [N=4,638]

|  | | Physical frailty | | | Psychological frailty | Social frailty |
| --- | --- | --- | --- | --- | --- | --- |
|  |  | 4 indicators | 3 indicators including exhaustion | 3 indicators including weight loss |  |  |
| Physical frailty | 4 indicators | **1.00** | **1.00** | **0.90** | 0.41 | 0.16 |
|  | 3 indicators including exhaustion | **1.00** | **1.00** | **0.89** | 0.40 | 0.16 |
|  | 3 indicators including weight loss | **0.90** | **0.89** | **1.00** | 0.29 | 0.12 |
| Psychological frailty | | 0.41 | 0.40 | 0.29 | 1.00 | - |
| Social frailty | | 0.16 | 0.16 | 0.12 | - | 1.00 |

Table 9: Measurement model for three specifications of physical frailty and using confirmatory factor analysis (CFA) using maximum likelihood with robust standard errors (MLR) compared with weighted least squares with mean- and variance-adjustment (WLSMV) [N = 4,547 for first two specifications; N=4,440 for the third specification]

| **Physical frailty specification** | **Standardized coefficient (standard error)** | |
| --- | --- | --- |
|  | **MLR** | **WLSMV** |
| **Four indicators:**  Slowness ^a^  Weakness ^b^  Exhaustion ^c^  Weight loss ^d^ | 0.76 (0.03)  0.54 (0.02)  0.55 (0.02)  0.28 (0.03) | 0.74 (0.02)  0.54 (0.02)  0.58 (0.02)  0.28 (0.03) |
| **Three indicators including exhaustion:**  Slowness ^a^  Weakness ^b^  Exhaustion ^c^ | 0.78 (0.03)  0.52 (0.02)  0.54 (0.03) | 0.76 (0.02)  0.53 (0.02)  0.57 (0.02) |
| **Three indicators including weight loss:**  Slowness ^a^  Weakness ^b^  Weight loss ^d^ | 0.60 (0.05)  0.68 (0.05)  0.30 (0.03) | 0.58 (0.05)  0.70 (0.05)  0.29 (0.03) |

P-values are <0.05 for all coefficients

^a^ Slowness: mean gait speed multiplied by a factor of -1

^b^ Weakness: dominant hand grip strength multiplied by a factor of -1 (males) or -1.5 (females)

^c^ Exhaustion: positive response to either or both of two items of CES-D scale on “could not get going much of the time in the past week” and “felt everything they did during the past week was an effort”

^d^ Weight loss: decrease in weight of more than 5 kg from wave 0 to wave 2

Table 10: Linear regression of chronic disease, functional status, self-rated health on factor scores, and Frailty Index for three physical frailty specifications adjusted for age using maximum likelihood with robust standard errors (MLR) compared with weighted least squares with mean- and variance-adjustment (WLSMV): standardized coefficients (95% confidence interval) [N=4,638 for all, 2,070 for male, and 2,568 for female]

| Physical frailty specifications | Number of chronic diseases | | Number of basic and instrumental activities of daily living items performed with difficulty | | Categories of poor self-rated health | | Frailty Index | |
| --- | --- | --- | --- | --- | --- | --- | --- | --- |
|  | MLR | WLSMV | MLR | WLSMV | MLR | WLSMV | MLR | WLSMV |
| 4 indicators | 0.35  (0.32 to 0.38) | 0.35  (0.32 to 0.38) | 0.49  (0.46 to 0.52) | 0.49  (0.46 to 0.52) | 0.51  (0.48 to 0.53) | 0.50  (0.48 to 0.53) | 0.76  (0.73 to 0.79) | 0.76  (0.73 to 0.79) |
| 3 indicators (including exhaustion) | 0.34  (0.31 to 0.37) | 0.34  (0.31 to 0.37) | 0.48  (o.46 to 0.51) | 0.48  (0.45 to 0.51) | 0.50  (0.47 to 0.53) | 0.50  (0.47 to 0.53) | 0.75  (0.72 to 0.78) | 0.75  (0.72 to 0.78) |
| 3 indicators (including weight loss) | 0.30  (0.27 to 0.33) | 0.29  (0.26 to 0.32) | 0.39  (0.36 to 0.43) | 0.38  (0.35 to 0.41) | 0.40  (0.37 to 0.43) | 0.39  (0.36 to 0.42) | 0.62  (0.59 to 0.65) | 0.61  (0.57 to 0.64) |

Table 11: Correlation of factor scores for three physical frailty specifications with those for psychological and social frailty using maximum likelihood with robust standard errors (MLR) compared with weighted least squares with mean- and variance-adjustment (WLSMV): Pearson’s coefficient (95% confidence interval) [N=4,638 for all, 2,070 for male, and 2,568 for female]

| Physical frailty specifications | Psychological frailty | | Social frailty | |
| --- | --- | --- | --- | --- |
|  | MLR | WLSMV | MLR | WLSMV |
| 4 indicators | 0.41  (0.38 to 0.43) | 0.41  (0.38 to 0.43) | 0.16  (0.13 to 0.20) | 0.16  (0.13 to 0.20) |
| 3 indicators (including exhaustion) | 0.40  (0.37 to 0.43) | 0.40  (0.37 to 0.43) | 0.16  (0.13 to 0.19) | 0.16  (0.13 to 0.19) |
| 3 indicators (including weight loss) | 0.29  (0.26 to 0.32) | 0.29  (0.26 to 0.31) | 0.12  (0.08 to 0.15) | 0.12  (0.08 to 0.15) |

Figure 3: Distribution of factor scores for the three physical frailty specifications [N = 4,547 for first two specifications; N=4,440 for the third specification]
